# Rapid Microbial Quality Assessment of Chicken Liver Inoculated or Not With *Salmonella* Using FTIR Spectroscopy and Machine Learning

**DOI:** 10.3389/fmicb.2020.623788

**Published:** 2021-02-04

**Authors:** Dimitra Dourou, Athena Grounta, Anthoula A. Argyri, George Froutis, Panagiotis Tsakanikas, George-John E. Nychas, Agapi I. Doulgeraki, Nikos G. Chorianopoulos, Chrysoula C. Tassou

**Affiliations:** ^1^Institute of Technology of Agricultural Products, Hellenic Agricultural Organization DIMITRA, Athens, Greece; ^2^Laboratory of Food Microbiology and Biotechnology, Department of Food Science and Human Nutrition, School of Food and Nutritional Sciences, Agricultural University of Athens, Athens, Greece

**Keywords:** chicken liver, poultry, spoilage, *Salmonella*, Fourier-transform infrared spectroscopy, machine learning, support vector regression

## Abstract

Chicken liver is a highly perishable meat product with a relatively short shelf-life and that can get easily contaminated with pathogenic microorganisms. This study was conducted to evaluate the behavior of spoilage microbiota and of inoculated *Salmonella enterica* on chicken liver. The feasibility of Fourier-transform infrared spectroscopy (FTIR) to assess chicken liver microbiological quality through the development of a machine learning workflow was also explored. Chicken liver samples [non-inoculated and inoculated with a four-strain cocktail of *ca*. 10^3^ colony-forming units (CFU)/g *Salmonella*] were stored aerobically under isothermal (0, 4, and 8°C) and dynamic temperature conditions. The samples were subjected to microbiological analysis with concomitant FTIR measurements. The developed FTIR spectral analysis workflow for the quantitative estimation of the different spoilage microbial groups consisted of robust data normalization, feature selection based on extra-trees algorithm and support vector machine (SVM) regression analysis. The performance of the developed models was evaluated in terms of the root mean square error (RMSE), the square of the correlation coefficient (*R*^2^), and the bias (B*_f_*) and accuracy (A*_f_*) factors. Spoilage was mainly driven by *Pseudomonas* spp., followed closely by *Brochothrix thermosphacta*, while lactic acid bacteria (LAB), *Enterobacteriaceae*, and yeast/molds remained at lower levels. *Salmonella* managed to survive at 0°C and dynamic conditions and increased by *ca.* 1.4 and 1.9 log CFU/g at 4 and 8°C, respectively, at the end of storage. The proposed models exhibited A*_f_* and B*_f_* between observed and predicted counts within the range of 1.071 to 1.145 and 0.995 to 1.029, respectively, while the *R*^2^ and RMSE values ranged from 0.708 to 0.828 and 0.664 to 0.949 log CFU/g, respectively, depending on the microorganism and chicken liver samples. Overall, the results highlighted the ability of *Salmonella* not only to survive but also to grow at refrigeration temperatures and demonstrated the significant potential of FTIR technology in tandem with the proposed spectral analysis workflow for the estimation of total viable count, *Pseudomonas* spp., *B. thermosphacta*, LAB, *Enterobacteriaceae*, and *Salmonella* on chicken liver.

## Introduction

Meat is considered as the most nutritious and energy-rich food product that can provide the human body with all the essential amino acids and micronutrients needed for growth and development (Wood, 2017; Ahmad et al., 2018). Chicken meat has been evidenced to be of greater benefit for human health than red meat because of comparably higher contents of proteins as well as lower contents of fat and cholesterol (Probst, 2009; Pereira and Vicente, 2013; Wood, 2017). Among the edible components of chicken, giblets and especially livers are widely consumed in many countries throughout the world due to their low cost/price, high nutritional value, and short preparation time (Álvarez-Astorga et al., 2002; Shenoda et al., 2019). Chicken liver is an excellent source of important nutrients such as proteins, vitamins (e.g., A, B1, B3, B5, and B6), essential amino acids, and minerals (e.g., Fe, Cu, Mn, and Zn), which are sometimes at levels higher compared to muscle tissue (Jokanović et al., 2014; Seong et al., 2015). The rich nutritional composition along with the neutral pH and high water activity render the chicken liver highly perishable due to microbial growth (Nychas et al., 2008; Odeyemi et al., 2020).

Bacterial contaminants can be introduced at multiple stages along the food chain, including production, slaughter, processing, handling, storage, and preparation, leading thus to significant economic losses (Silva, 2013; Rouger et al., 2017b). The genera of *Pseudomonas*, *Brochothrix*, lactic acid bacteria, and *Enterobacteriaceae* are considered as potential spoilers of chicken meat during storage at low temperatures (Doulgeraki et al., 2012; Rouger et al., 2017b). Chicken microbiota may also harbor pathogenic species, with *Salmonella* being the most important zoonotic agent responsible for human gastroenteritis due to poultry meat consumption [EFSA (European Food Safety Authority) and ECDC (European Centre for Disease Prevention and Control), (2017); EFSA Panel on Biological Hazards et al., 2019]. Several reports of salmonellosis outbreaks associated with chicken liver consumption have been released worldwide, including the USA (Lanier et al., 2018) and EU [EFSA (European Food Safety Authority) and ECDC (European Centre for Disease Prevention and Control), (2017)]. At the EU level (2014–2016), *Salmonella* strains were responsible for more than 753 strong-evidence foodborne outbreaks, with 46 outbreaks attributed to broiler meat and products thereof (EFSA Panel on Biological Hazards et al., 2019). Moreover, *Salmonella* prevalence on chicken giblets has been reported to be over 53.4% in Greece (Zdragas et al., 2012), 59.4% in the mid-Atlantic region of the United States (Jung et al., 2019), and 4.8% in Argentina (Procura et al., 2019). Given the increased production of chicken meat in the last decade, ensuring the microbial safety and quality of chicken liver is of primary importance (Augère-Granier, 2019).

So far, the freshness, spoilage, or safety of meat and poultry products has been relying on sensory, microbiological, and chemical analyses on the finished product (European Commission, 2005). Sensory methods require highly trained personnel, which is costly and not convenient for routine analyses. On the other hand, chemical as well as microbiological analyses (conventional or molecular) are time-consuming, laborious, and destructive to the test products, with some of them requiring high-tech tools and providing retrospective results (Nychas et al., 2008). Thus, their potential to be used for on-, in-, or at-line monitoring in the food industry is limited (Nychas et al., 2016). Exploration of various analytical tools for rapid, non-invasive, and non-destructive quantitative assessment of safety and quality characteristics presents a scientific challenge given the importance of microbiological spoilage and safety on the deterioration of chicken liver freshness.

Nowadays, many different sensors, such as near-infrared spectroscopy, Fourier-transform infrared spectroscopy (FTIR), Raman spectroscopy, hyperspectral and multispectral imaging, have been employed to evaluate freshness, microbial quality, and adulteration of foods (Panagou et al., 2011; Alexandrakis et al., 2012; Argyri et al., 2014; He and Sun, 2015; Tsakanikas et al., 2015; Rateni et al., 2017; Ropodi et al., 2018; Keshavarzi et al., 2020). FTIR spectroscopy, a biochemical fingerprinting technique, in conjunction with chemometrics, machine learning, or computational intelligence methods, has shown significant potential in providing information related to food safety and quality of meat and poultry (Ellis et al., 2004; Ammor et al., 2009; Papadopoulou et al., 2011; Argyri et al., 2013; Ropodi et al., 2016; Pavli et al., 2018; Rahman et al., 2018). However, a repeated challenge that researchers often have to face is the choice of the machine learning approach in order to handle the complex, multivariate nature of the FTIR sensor output. This usually incorporates the analysis of various regression algorithms for reduction of FTIR data dimensionality to ultimately obtain accurate and reliable predictions (Torrione et al., 2014; Ropodi et al., 2016; Tsakanikas et al., 2016, 2020).

Limited research data on the microbiological quality of chicken liver are available nowadays, while studies on the potential of *Salmonella* to survive and/or proliferate on chicken liver during extended refrigerated storage are even rarer. Hasapidou and Savvaidis (2011) evaluated the effect of oregano essential oil and ethylenediaminetetraacetic acid chelator on the quality characteristics of chicken liver stored under refrigerated (4°C) modified atmosphere conditions. Papazoglou et al. (2012) investigated the effect of thyme oil on the quality of vacuum-packaged chicken liver stored under refrigeration (4°C). Recently, Jung et al. (2019) quantified the levels of *Salmonella* onto and into raw chicken liver following extended refrigerated (4°C) or frozen (−20°C) storage. Moreover, despite the extended implementation of FTIR spectroscopy to various plant and animal food commodities, to our knowledge, there is limited, if any, information available on chicken liver. On this respect, the present work was attempted (1) to record spoilage microbiota on chicken liver stored aerobically at isothermal and dynamic temperature conditions alone and in the presence of inoculated *Salmonella* (2) to monitor the behavior of *Salmonella* on chicken liver under the same storage conditions, and (3) to quantitatively assess spoilage on chicken liver based on FTIR spectral data and microbiological counts from non-inoculated and inoculated-with-*Salmonella* samples through the development of a spectral analysis and prediction model building workflow that will be specific to chicken liver.

## Materials and Methods

### Sample Preparation and Experimental Design

Fresh chicken giblets were obtained from a local industry on the day of production and transported (within 30 min) under refrigeration to the laboratory. The chicken livers (*ca*. 50 ± 2 g) were then aseptically removed from the giblets and packed aerobically in duplicate in styrofoam trays that were subsequently wrapped manually with air-permeable polyethylene plastic film (non-inoculated samples). In parallel, chicken livers (*ca*. 50 ± 2 g) were inoculated with *Salmonella enterica subsp. enterica* serovar Enteritidis (four-strain cocktail) and packed under the same conditions. The packed livers were stored under controlled isothermal conditions (0, 4, and 8°C) and dynamic temperature conditions (0, 4, and 8°C every 8 h) for up to 10 days in a high-precision (±0.1°C) cooled incubator (IC 150-R, Agrolab, Capri, Italy). The latter conditions were selected on the basis to simulate the chicken livers’ temperature fluctuations in chill chain. In total, two chronically independent experiments (different product batches) were performed for every treatment (non-inoculated and inoculated samples) and storage temperature, with duplicate samples analyzed microbiologically at each time interval (*n* = 4).

### Inoculation of Chicken Liver With *Salmonella*

A four-strain cocktail of *Salmonella enterica subsp. enterica* serovar Enteritidis (FMCC B56 PT4, FMCC B-57 PT7, B64, and ATCC 13076) was used for the inoculation of chicken livers. The strains were kindly provided by Prof. Nychas G-J.E, Lab of Microbiology and Biotechnology, Agricultural University of Athens, Athens, Greece. The *Salmonella* strains were maintained at −80°C, were revived at 10 ml brain heart infusion (BHI, LAB M, LAB049) broth after overnight incubation at 37°C, and were subcultured in 10 ml fresh BHI broth (18 h, 37°C). Cells of the individual cultures were then harvested by centrifugation (5,000 × *g*, 10 min, 4°C) and washed twice in 10 ml Ringer’s solution (¼ strength, LAB M). The washed cells of each strain were resuspended in 10 ml Ringer’s solution and were combined in equal volumes to generate a four-strain cocktail. The chicken liver samples were separately inoculated with 50 μl of appropriate dilution of the pathogen cocktail and were then held for approximately 10 min at ambient temperature (21 ± 2°C) to allow the inocula to attach to the surface. The livers were then inverted with sterile tweezers, and the process was repeated on the opposite side to yield a final population of the pathogen of *ca*. 10^3^ colony-forming units (CFU)/g.

### Microbiological Analyses

The chicken liver samples (20 g) were separately weighted aseptically in a sterile stomacher bag containing ¼ strength Ringer’s solution (40 ml) and stomached for 60 s at room temperature (Stomacher 400 Circulator, Seward Limited, Norfolk, United Kingdom). Appropriate serial dilutions of the resulting homogenate were spread (0.1 ml) or poured (1 ml) on different selective and non-selective agar plates for the enumeration of the following bacterial groups: total viable count (TVC) on Tryptic soy agar (REF 4021502, Biolife) incubated at 30°C for 2–3 days, lactic acid bacteria (LAB) on de Man-Rogosa-Sharp medium (LAB233, LABM) overlaid with the same medium and incubated at 30°C for 3–5 days, *Brochothrix thermosphacta* on streptomycin thallous acetate actidione agar (REF 4020792 with the addition of antibiotic REF 4240052, Biolife) incubated at 25°C for 2 days, yeasts/molds on rose bengal chloramphenicol agar (BK151HA, Biokar) incubated at 25°C for 2–5 days, *Enterobacteriaceae* on violet red bile glucose agar (CM 0485, Oxoid) overlaid with the same medium and incubated at 37°C for 24 h, *Pseudomonas* spp. on *Pseudomonas* agar base (LAB108 supplemented with selective supplement cetrimide fucidin cephaloridine, Modified C.F.C X108, LABM) incubated at 25°C for 2 days, and *Salmonella* on xylose lysine deoxycholate (LAB032, LABM) incubated at 37°C for 16–18 h. The plates for each agar medium were examined visually for typical morphological characteristics of colonies. Additionally, the selectivity of growth media was checked by Gram staining and microscopic examination of smears prepared from randomly selected colonies obtained from all media.

Uninoculated chicken liver samples (three liver samples/batch) were also analyzed at the beginning of storage using enrichment method for the detection of *Salmonella* that could naturally appear on chicken liver (ISO 6579-1:2017).

### pH Measurements

The pH value was monitored using a digital pH meter (HI 2211 pH-ORP Meter, HANNA Instruments, United States) after the end of microbiological analyses by immersing the glass electrode into the homogenate.

### FTIR Spectroscopy

FTIR analysis was performed on the surface of chicken liver samples at 25°C with a ZnSe 45° attenuated total reflectance flat plate crystal on a Perkin Elmer Frontier FTIR spectrometer equipped with DLaTGS detector with a KBr window. The spectrometer was equipped with software PerkinElmer Spectrum v10.4.2 to collect spectra over the wavelength range of 4,000 to 650 cm^−1^. Scans per measurement were four, with a resolution of 4 cm^−1^. Prior to the measurement of the tested samples, reference (background) spectra were acquired using the cleaned blank (no added liver sample) crystal. The tested samples were transferred to the crystal plate and then pressed with a gripper to ensure the best possible contact with the surface of the crystal. After each measurement, the crystal’s surface was cleaned, initially with detergent and distilled water and then with ethanol, and dried using lint-free tissue. For each treatment (non-inoculated and inoculated samples stored at isothermal and dynamic storage conditions) and time interval, three FTIR spectra were acquired for each of the two biological replicates of the experiment (*n* = 12). A total of 878 FTIR spectra were collected and used for further analyses (*n* = 442 for non-inoculated samples and *n* = 436 for inoculated-with-*Salmonella* samples). The FTIR spectra that were ultimately used in further analyses were in the approximate wavelength range of 1,800 to 900 cm^−1^ as this spectral region has been shown to provide useful metabolic fingerprints with regard to meat spoilage (Papadopoulou et al., 2011; Argyri et al., 2013; Fengou et al., 2019).

### Mathematical Modeling of the Spectral Data

Three data sets from chicken liver samples stored at isothermal and dynamic temperature conditions, i.e., non-inoculated, inoculated with *Salmonella*, and their combination (i.e., non-inoculated and inoculated samples), were analyzed. For each of these data sets, the processing pipeline of acquired FTIR data consisted of a feature selection step (i.e., specific wavelengths/wavenumbers) on the bases of extra-trees regression ensemble (Geurts et al., 2006) followed by a support vector regression (SVR) of the different spoilage-related microbial groups (Smola and Scholkopf, 2004; Tsakanikas et al., 2016). Initially, prior to feature selection, the acquired FTIR spectra (*S*) were subjected to standard normal variate normalization under its robust version (Guo et al., 1999) according to:

$$S_{i}^{\mathrm{snv}}=\frac{S_{i}-\mathrm{median}\left(S\right)}{\mathrm{mad}\;\left(S\right)}$$

where *S_i_* and *S_i_*^snv^ is the *i*th spectrum and the corresponding normalized spectrum, respectively, and mad is the median absolute deviation, a robust measure of the variability of a univariate sample of quantitative data *s1*, *s2*, …, *sn* (Hoaglin et al., 2000) computed as:

$$\mathrm{mad}=\mathrm{median}\left(\left|S_{i}-\mathrm{median}\left(S\right)\right|\right)$$

The specific normalization scheme was selected on the basis that gives more reasonable (i.e., without artifacts) and enhanced quality data, eliminating the inherent multiplicative noise while reducing the correlated information along spectra (Tsakanikas et al., 2018). Then, prior to regression and to avoid overfitting of the dataset, due to the small number of samples compared to the large number of input variables, a feature selection step was introduced by applying the extremely randomized trees (extra-trees) algorithm (Geurts et al., 2006). The extra-trees algorithm is a tree-based ensemble method for reducing the dimensionality of spectral data which is characterized by high accuracy and computational efficiency. In this context, the variable set was reduced by preserving the critical features that best represent the samples (in terms of predicting the inherent microbial load) and excluding all the expendable ones (Tsakanikas et al., 2018). Following the feature selection, SVR (Smola and Scholkopf, 2004) was applied to the reduced dataset for the estimation/prediction of the microbial populations from the corresponding spectroscopic data. SVM/R is a robust supervised tool for both classification and regression (Vapnik et al., 1997) and has been used in various food quality applications (Du et al., 2007; Argyri et al., 2013, 2014; Schmutzler et al., 2015; Estelles-Lopez et al., 2017; Ropodi et al., 2018; Yu et al., 2019; Fengou et al., 2020; Tsakanikas et al., 2020). Briefly, in SVMs, the original data x are mapped from the input space onto a high-dimensional feature space *via* a non-linear mapping function (kernel function) in order to construct an optimal hyperplane that minimizes the total square distance to all data points. In this study, the radial kernel (radial basis function) was used for fitting FTIR data. Grid search for the optimal cost (*C*) and gamma (*γ*) parameters, coupled with 10-fold cross-validation, was employed for model development and parameter optimization (Pedregosa et al., 2011).

In order to generate the predictive models, the pretreated dataset was randomly partitioned (using a random generator) over 50 iterations into a training (calibration) dataset, which contained 70% of the samples, and a test dataset composed of the remaining samples for external validation. The training dataset was used for calibration, i.e., model building, while the test (validation) dataset was used to externally evaluate the performance of such model. Samples stored at both isothermal and dynamic temperature conditions were equally represented in training and testing datasets in order to generate a realistic prediction model based on real-life conditions. Data pre-treatment, featuring selection, model development, and validation were implemented using Python 3.6 and scikit-learn library (Pedregosa et al., 2011).

### Model Validation

The performance of the regression models was quantified by the calculation of the root mean square error (RMSE), the square of the correlation coefficient (*R*^2^), the bias (B*_f_*) and accuracy (A*_f_*) factors (Ross, 1996), and the accuracy of prediction (Mohareb et al., 2016; Estelles-Lopez et al., 2017). The RMSE quantifies the average deviation between predicted and observed values (i.e., the smaller the value of the RMSE, the closer the predicted values are to the observed values). The A*_f_* provides a measure of how close predictions are to observations. An A*_f_* = 1 indicates a perfect agreement between observed and predicted values. The B*_f_* gives a measure of systematic under- or over-prediction by the model. A B*_f_* = 1 indicates a perfect agreement between predictions and observations, while a B*_f_* < 1 indicates that a growth model is fail-safe (i.e., predicted values are smaller than observed values, giving a margin of safety). The accuracy of prediction provides a measure of the percentage of samples correctly predicted (i.e., difference between predicted and observed value is <1) out of the total number of the samples within the dataset.

### Statistical Analysis

Differences in microbial populations between non-inoculated and inoculated-with-*Salmonella* samples (2 batches, *n* = 2 per batch) were tested with ANOVA. Significance was established at *p* < 0.05. Data analysis was carried out with SPSS (IBM SPSS Statistics for Windows, Version 26.0. Armonk, NY: IBM Corp.).

## Results and Discussion

### Population Dynamics on Non-inoculated Chicken Liver

**Figure 1:**
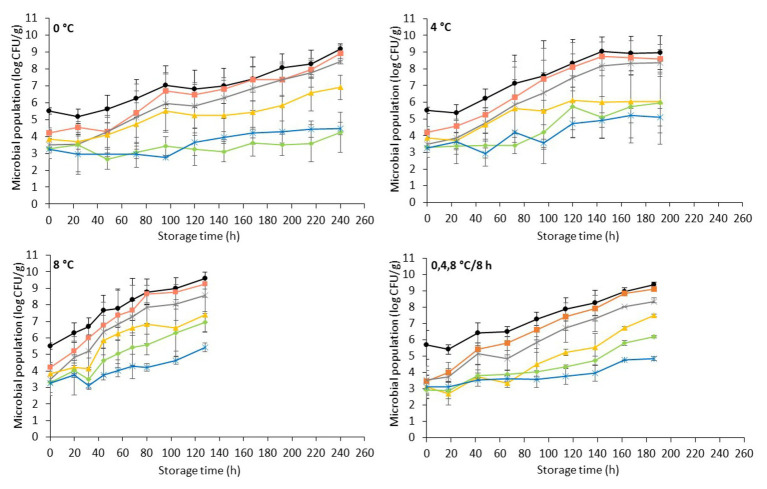
Evolution of indigenous spoilage microbiota (mean ± standard deviation, *n* = 4) on chicken liver during aerobic storage at different isothermal (0, 4, and 8°C) and dynamic temperature conditions (0, 4, and 8°C every 8 h). Total viable count (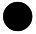), *Pseudomonas* spp. (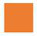), *Brochothrix thermosphacta* (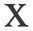), lactic acid bacteria (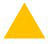), *Enterobacteriaceae* (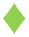), and yeast/molds (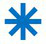).

The evolution of the spoilage microbiota (mean ± standard deviation, *n* = 4) on non-inoculated chicken liver stored aerobically at isothermal (0, 4, and 8°C) and dynamic temperature conditions (0, 4, and 8°C every 8 h) is presented in Figure 1. The initial (day 0) level of TVC on chicken liver was 5.6 ± 0.6 log CFU/g, which is in accordance with previous studies (Hasapidou and Savvaidis, 2011; Papazoglou et al., 2012; Jung et al., 2019). Various initial bacterial loads (2.0–6.3 log CFU/g) have been reported for other animal livers (Shelef, 1975; Gill and DeLacy, 1982; Hanna et al., 1982; Woolthuis et al., 1984; Rivas et al., 1992; Hernández-Herrero et al., 1999; Devatkal et al., 2004; Fernández-López et al., 2006; Silva et al., 2020). The elevated microbiological load observed in fresh liver could be attributed to cross-contamination during slaughter and fabrication (Silva, 2013; Rouger et al., 2017b).

The microbial association of chicken liver consisted mainly of *Pseudomonas* spp. (3.8 ± 0.7 log CFU/g), *B. thermosphacta* (3.5 ± 0.5 log CFU/g), LAB (3.5 ± 0.7 log CFU/g), *Enterobacteriaceae* (3.1 ± 0.5 log CFU/g), and yeasts/molds (3.2 ± 0.6 log CFU/g). The aerobic storage of chicken livers generally allowed the growth of microorganisms at high levels, with *Pseudomonas* spp. being the dominant spoilage microorganism, followed closely by *B. thermosphacta*, while LAB, *Enterobacteriaceae*, and yeasts/molds remained at lower levels at both isothermal and dynamic temperature conditions (Figure 1). The microbial profile described above is in accordance with other studies on aerobically stored chilled chicken liver and other poultry products (Balamatsia et al., 2007; Papazoglou et al., 2012; Rouger et al., 2017a; Lytou et al., 2018). The dominance of *Pseudomonas* spp. on aerobically stored red meat (Nychas et al., 2008; Ercolini et al., 2011; Pennacchia et al., 2011; Doulgeraki and Nychas, 2013) and poultry (Mellor et al., 2011; Sahar and Dufour, 2014; Vasconcelos et al., 2014; Lytou et al., 2018; Rahman et al., 2018; Saenz-García et al., 2020) is well documented. *B. thermosphacta*, a Gram-positive fermentative organism, has been recognized as the dominant spoilage species along with LAB on modified-atmosphere- and vacuum-stored meats (Kakouri and Nychas, 1994; Nychas et al., 2008; Doulgeraki et al., 2010; Ercolini et al., 2011; Pennacchia et al., 2011; Gribble and Brightwell, 2013). However, due to its ubiquitous nature and in agreement with the results of this study, it may also play an important role in shortening the shelf-life of aerobically stored chicken meat (Mikš-Krajnik et al., 2016; Lytou et al., 2018). The rest of the facultative anaerobic bacteria, LAB and *Enterobacteriaceae*, grew to similar levels in the final stages of storage at 4 and 8°C, while at 0°C LAB outgrew *Enterobacteriaceae*. Similar growth patterns have been observed by Vasconcelos et al. (2014) in poultry breast fillets stored at 3 and 7°C. In contrast, Papazoglou et al. (2012) reported lower levels for *Enterobacteriaceae* compared to LAB after storage of chicken livers at 4°C, while Rivas et al. (1992) reported lower levels for LAB on lamb liver stored at 0 and 3°C. The yeasts/mold levels appeared to be lower than the rest of the bacterial populations, in accordance with previous studies reporting yeasts and molds to be a minor part of the microbial association of poultry products, thus contributing less to poultry spoilage (Dillon and Board, 1991; Ismail et al., 2000; Balamatsia et al., 2007). As expected, the dynamics of these populations and their contribution to the final microbiota and, consequently, the spoilage process were temperature dependent, with the growth rate of the different microbial groups being progressively higher with increasing storage temperature (Nychas et al., 2008; Vasconcelos et al., 2014; Galarz et al., 2016).

It has to be noted that, during storage, chicken liver samples presented substantial inter-batch variability with regards to the levels of the different microbial groups (i.e., lower microbial counts in the first compared to the second batch) at all storage temperatures and most of the sampling points (data not shown). This variability could be attributed to factors such as the intrinsic characteristics of the liver tissue and the hygienic practices during slaughter and handling which seem to affect the structure of the indigenous microbial community and its evolution during storage (Huis In’t Veld, 1996; Nychas et al., 2008; Tougan et al., 2013; Luong et al., 2020; Odeyemi et al., 2020). As a result, microbiological spoilage on chicken liver (i.e., TVC > 7 log CFU/g; Nychas and Tassou, 1997; Mikš-Krajnik et al., 2016; Rouger et al., 2017b) was attained at 192 and 72 h of storage at 0°C for the first and second batch, respectively, at 120 and 72 h at 4°C and at 68 and 32 h at 8°C (data not shown). By taking this variability into account (i.e., averaging of TVC populations from both batches), chicken liver spoilage was evident at 144, 72, and 44 h of storage at 0, 4, and 8°C, respectively, and at 90 h of storage at dynamic temperatures (Figure 1). Similar results have been observed in the study by Hasapidou and Savvaidis (2011) who reported a shelf-life of 3 days for refrigerated (4°C) chicken liver.

Visible deterioration of chicken liver samples, i.e., surface browning and appearance of visible colonies, in most cases agreed with microbiological spoilage (TVC > 7 log CFU/g), while the onset of off odors was evident at later time intervals. Our observation is in agreement with the findings of Rivas et al. (1992) and Gill and DeLacy (1982) for lamb and sheep liver, respectively. The malodorous volatiles in high-protein meats are associated with microbial degradation of nitrogenous compounds, such as amino acids, and are usually the first indication of spoilage. Pseudomonads, which are in most cases responsible for the aerobic spoilage of meat, show a preference in utilizing simple carbohydrates (i.e., glucose) prior to free amino acids (Nychas et al., 2008). In the case of liver, which is characterized by high contents of glucose throughout storage, the preference of spoilage organisms for glucose allowed the formation of visible colonies on the surface of liver before the off odors’ accumulation (Gill and DeLacy, 1982; Rivas et al., 1992).

**Figure 2:**
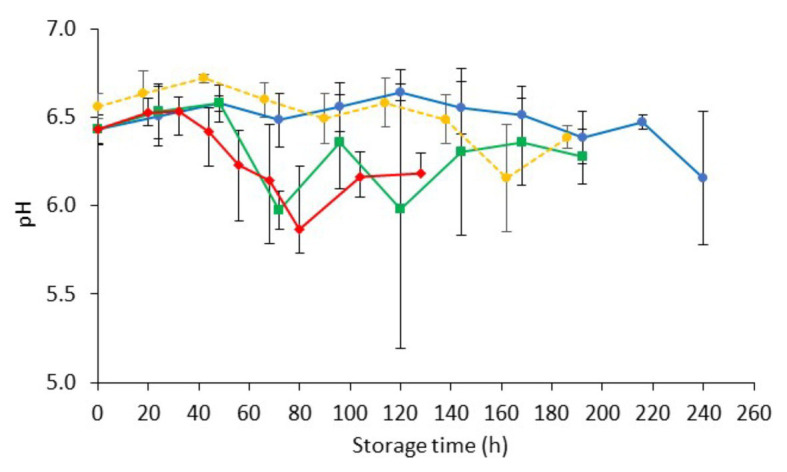
Changes on pH values (mean ± standard deviation, *n* = 4) of non-inoculated chicken liver during aerobic storage at different isothermal (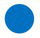 0, 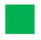 4, and 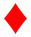 8°C) and dynamic temperature conditions (
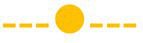
 0, 4, and 8°C every 8 h).

The pH of fresh chicken liver (day 0) was 6.50 ± 0.10, which is in agreement with previous studies (Hasapidou and Savvaidis, 2011; Papazoglou et al., 2012; Figure 2). Similar pH values (ranging from 6.15 to 6.84) have been reported for fresh beef, pork, lamb, buffalo, ostrich, and pork livers (Hanna et al., 1982; Hernández-Herrero et al., 1999; Devatkal et al., 2004; Fernández-López et al., 2006; Custódio et al., 2016). The pH of chicken liver fluctuated during storage but remained relatively constant throughout (minor reductions ranging from 0.15 to 0.28 units for the different storage temperatures). This result is in line with previous studies in aerobically stored animal livers (Gill and DeLacy, 1982; Hanna et al., 1982; Woolthuis et al., 1984; Papazoglou et al., 2012).

### Population Dynamics on Chicken Liver Inoculated With *Salmonella*

**Figure 3:**
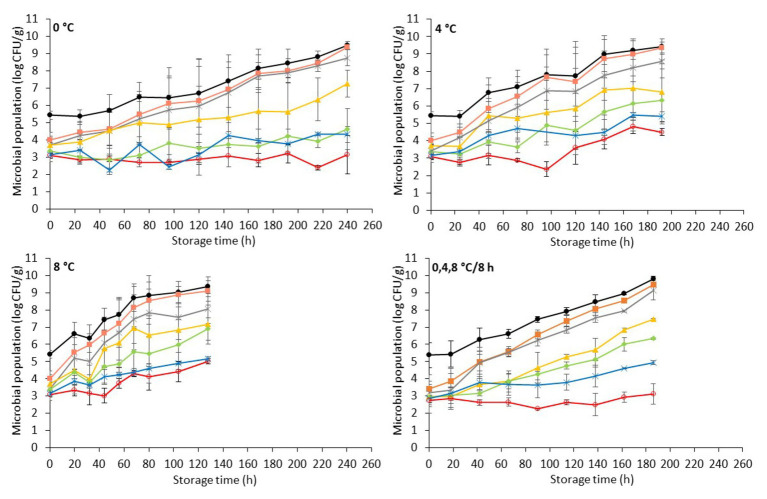
Evolution of *Salmonella* and indigenous spoilage microbiota (mean ± standard deviation, *n* = 4) on chicken liver during aerobic storage at different isothermal (0, 4, and 8°C) and dynamic temperature conditions (0, 4 and 8°C every 8 h). Total viable count (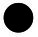), *Pseudomonas* spp. (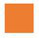), *Brochothrix thermosphacta* (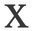), lactic acid bacteria (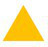), *Enterobacteriaceae* (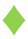), yeast/molds (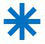), and *Salmonella* (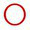).

The changes of the indigenous spoilage microbiota and *Salmonella* (mean ± standard deviation, *n* = 4) on chicken liver inoculated with the pathogen during aerobic storage at isothermal (0, 4, and 8°C) and dynamic temperature conditions (0, 4, and 8°C every 8 h) are presented in Figure 3. The dynamics of the spoilage bacterial populations were influenced by the storage temperature but not (*p* > 0.05) by the presence of the pathogen *per se* compared to the respective populations in non-inoculated liver (Figure 1 and Figure 3).

Similarly, the population dynamics of *Salmonella* was significantly influenced by the storage temperature. Specifically, *Salmonella* remained at inoculation levels (2.9 ± 0.2 log CFU/g) during storage at 0°C (240 h), while at 4°C it presented a substantial increase after 96 h, reaching final populations of 4.5 ± 0.2 log CFU/g at 192 h. At 8°C, the pathogen increased after 44 h, reaching 5.0 ± 0.2 log CFU/g at the end of the storage period (128 h). However, *Salmonella’s* onset of growth at 4 and at 8°C coincided chronically with the microbiological spoilage (TVC > 7 log CFU/g) of chicken liver (Figure 3). At dynamic temperature conditions, the *Salmonella* levels remained practically unaffected during aerobic storage for 186 h (Figure 3). Salmonellae growth, most of the times, is prevented at chilled temperatures (Airoldi and Zottola, 1988; Rhoades et al., 2013; Lerasle et al., 2014). In the study by Oscar (2011), the researcher reported the survival of *Salmonella* Typhimurium at temperatures between 4 and 8°C on chicken skin. In addition, in the recent study by Jung et al. (2019), *Salmonella* inoculated either into or onto chicken liver was shown to decrease by *ca*. 1 log CFU/g after 2 days of storage at 4°C. However, in agreement with the results of the current study, salmonellae have been shown not only to survive but even grow on fresh meats, including minced chicken and chicken parts, stored at refrigerated temperatures (<10°C; Baker et al., 1986; D’Aoust, 1991; Smadi et al., 2012). Specifically, the growth of *S*. Typhimurium and *S.* Enteritidis has been displayed on minced meat and on chicken surfaces stored at 2°C within 1–6 days (Catsaras and Grebot, 1985; Baker et al., 1986). Kim et al. (2011) likewise reported the growth of *S.* Typhimurium at 5°C in kimbap, a Korean ready-to-eat food, while Kinsella et al. (2007) observed the survival and increased growth of a *S.* Typhimurium strain on beef after 72 h at 4°C. A possible explanation for the various growth patterns of *Salmonella* at low temperatures could be the different strains studied, the different types of raw material, and the variations on the levels and types of competing indigenous microbiota that could affect the survival or growth of *Salmonella* (Oscar, 2007; Silva et al., 2016).

**Figure 4:**
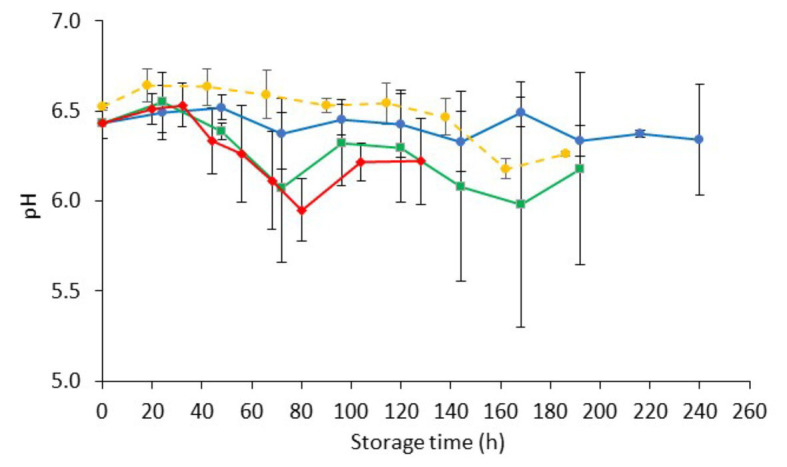
Changes on pH values (mean ± standard deviation, *n* = 4) of chicken liver inoculated with *Salmonella* during aerobic storage at different isothermal (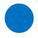 0, 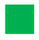 4, and 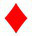 8°C) and dynamic temperature conditions (
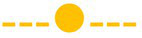
 0, 4, and 8°C every 8 h).

The initial pH (day 0) of inoculated-with-*Salmonella* chicken liver was 6.48 ± 0.08 (Figure 4). The storage of samples resulted in minor decreases in pH (ranging from 0.09 to 0.27, depending on the temperature) that were comparable to those in non-inoculated samples.

### FTIR Spectral Interpretation

**Figure 5:**
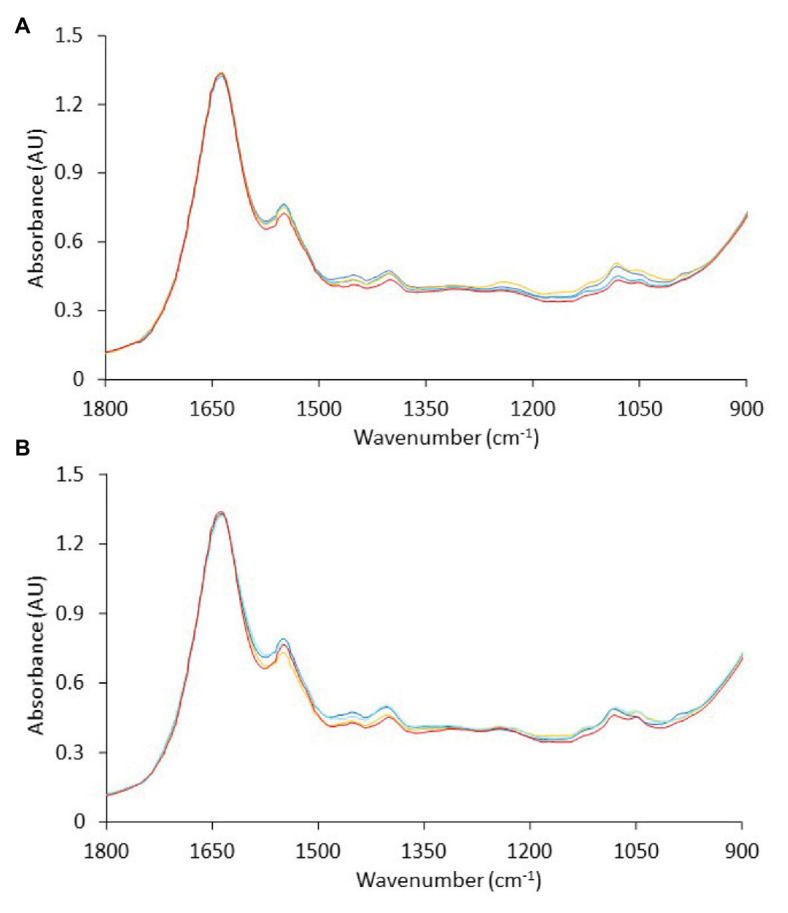
Representative Fourier-transform infrared spectra, in the wavenumber range of 1,800 to 900 cm^−1^, corresponding to **(A)** non-inoculated and **(B)** inoculated-with-*Salmonella* chicken liver samples at the beginning (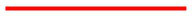, 0 h) and at the end of aerobic storage at 0°C (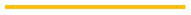, 240 h), 4°C (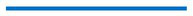, 192 h), and 8°C (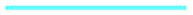, 128 h).

FTIR analyses were performed at each time point during storage of non-inoculated and inoculated-with-*Salmonella* chicken liver at different isothermal (0, 4, and 8°C) and dynamic (0, 4, and 8°C every 8 h) temperature conditions. Representative FTIR profiles, corresponding to non-inoculated and inoculated-with-*Salmonella* chicken liver samples at the beginning (fresh) and end (spoiled) of aerobic storage at different isothermal temperatures, are presented in Figure 5. Based on Figure 5, a major peak at 1,637 cm^−1^ was apparent in the chicken liver samples due to the presence of moisture (O–H stretch), with a contribution of amide I band of proteins (80% C=O stretch, 10% C–N stretch, and 10% C–N bend), whereas a second peak was observed at 1,549 cm^−1^ due to the absorbance of amide II band of proteins (40% C–N stretch, 60% N–H bend; Socrates, 2001; Ellis et al., 2002). Other minor peaks were observed at 1,473 cm^−1^ ascribed to lipids (CH_3_ asymmetric deformation, CH_3_ asymmetric bending, C–H deformation of CH_2_, CH_2_ scissoring vibration, C–H bending), with an underlying contribution of amines (asymmetric CH_3_ deformation vibration), at 1,453 cm^−1^ ascribed to fat (CH_2_ bending), at 1,402 cm^−1^ ascribed to amino acid side chains, lipids, and carbohydrates (C–H bend or C–O stretch in carboxylates) or nitro group (NO_2_ symmetric stretch), at 1,307 cm^−1^ attributed to amide III (30% C–N stretch, 30% N–H bend, 10% C=O–N bend, 20% others), at 1,243 cm^−1^ corresponding to lipids and nucleic acids (asymmetric PO_2_- stretch), with the contribution of amide III P=O stretch (30% C–N stretch, 30% N–H bend, 10% C=O–N bend, 20% others) and amines from free amino acids (C–N stretch), at 1,117 cm^−1^ ascribed to riboses (C–O stretch) and amines (NH_2_ rocking/twisting), at 1,081 cm^−1^ corresponding to nucleic acids and phospholipids (PO_2_ symmetric stretch)/C–O stretch, and finally at 1,044 cm^−1^ corresponding to lipids and polysaccharides (C–O, C–O–P stretch) (Socrates, 2001; Ellis et al., 2002, 2004; Böcker et al., 2007; Ammor et al., 2009).

**Figure 6:**
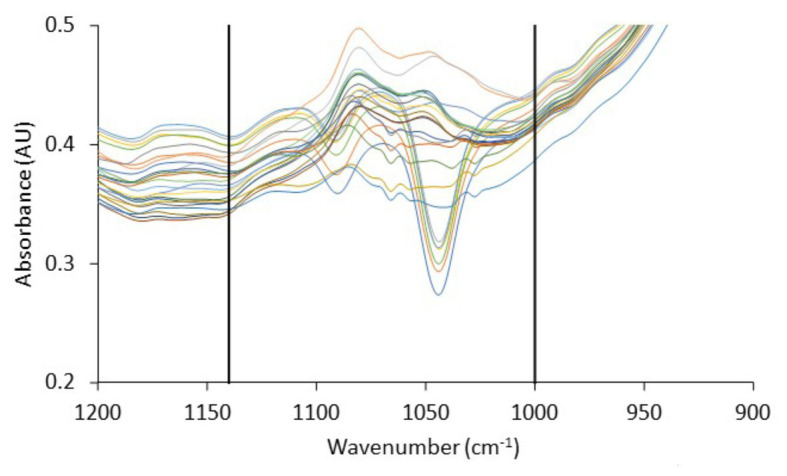
Fourier-transform infrared spectra, in the wavenumber range 1,200–900 cm^−1^, corresponding to fresh (0 h) chicken liver samples from the different batches used in the current study. Vertical lines delimit the wavenumber range (1,140–1,000 cm^−1^) in which significant inter- and intra-batch variability is observed.

The spectra presented generally similar patterns, with no profound feature peaks to reflect uniquely the difference in quality between fresh and spoiled chicken liver samples. However, features within this wavenumber area, commonly ascribed to amides and amines, are linked to microbiological and/or autolytic proteolysis of muscle meat proteins occurring during storage (Nychas and Tassou, 1997; Ellis et al., 2002; Alexandrakis et al., 2012; Vasconcelos et al., 2014; Fengou et al., 2019). At this point, it has to be noted that liver samples (fresh and spoiled) exhibited a substantial inter- and intra-batch variability in the approximate wavenumber range from 1,140 to 1,000 cm^−1^ (Figure 6), an area that is associated mostly with amines from free amino acids and strongly correlated to chicken spoilage (Ellis et al., 2002). Such variability could originate from differences in the intrinsic characteristics of the liver tissue as well as in the bacterial community structure and the associated biochemical changes on the surface of the chicken liver. Consequently, a machine learning approach was employed to analyze the spectra and quantify chicken liver spoilage along with FTIR data.

### Estimation of Chicken Liver Spoilage Using FTIR Data

FTIR spectroscopy in tandem with machine learning methodologies has been widely used in the literature for a rapid microbial quality assessment of foods (He and Sun, 2015). A major challenge in the field of machine learning that engineers have to overcome is the high dimensionality of spectral data which may negatively affect the performance of the models (Cai et al., 2018). In this study, to overcome this problem, we introduced an ensemble of feature selection based on extra-trees algorithm to select a subset of relevant and non-redundant features (Geurts et al., 2006). With this approach, features that had no and/or low correlation with the output variables, i.e., microbial populations, were reduced from 900 (wavelength 1,800–900 cm^−1^) to less than 110 (data not shown). With dimensionality reduction of FTIR data, the robustness and performance of the developed models would increase through the elimination of bias other than microbiological factors (e.g., bias attributed to inherent chicken liver samples and/or batch variability) and prevention of data overfitting (Tsakanikas et al., 2018).

Following dimensionality reduction, SVM radial regression models (Smola and Scholkopf, 2004) were developed for the correlation of spectral data with the population of selected microbial groups, namely, total viable counts, *Pseudomonas* spp., *B. thermosphacta*, LAB, *Enterobacteriaceae*, and *Salmonella*, on the surface of chicken liver samples. It should be noted that, in an effort to develop robust and realistic models, different sources of variability were incorporated in the analysis beyond the biological aspect (i.e., different batches). These included the biochemical fingerprint of *Salmonella* (Grewal et al., 2015) potentially present on chicken liver (i.e., datasets from inoculated-with-*Salmonella* liver alone or in combination with data from non-inoculated samples, in this study) as well as the various storage temperatures encountered in the cold chain [i.e., well-controlled refrigeration (0 and 4°C) to slightly abusive (8°C) and dynamic temperature conditions, in this study] and the resulting swifts in the structure of bacterial communities and microbial metabolites produced (Nychas et al., 2008; Doulgeraki et al., 2012; Tsakanikas et al., 2018, 2020).

**Figure 7:**
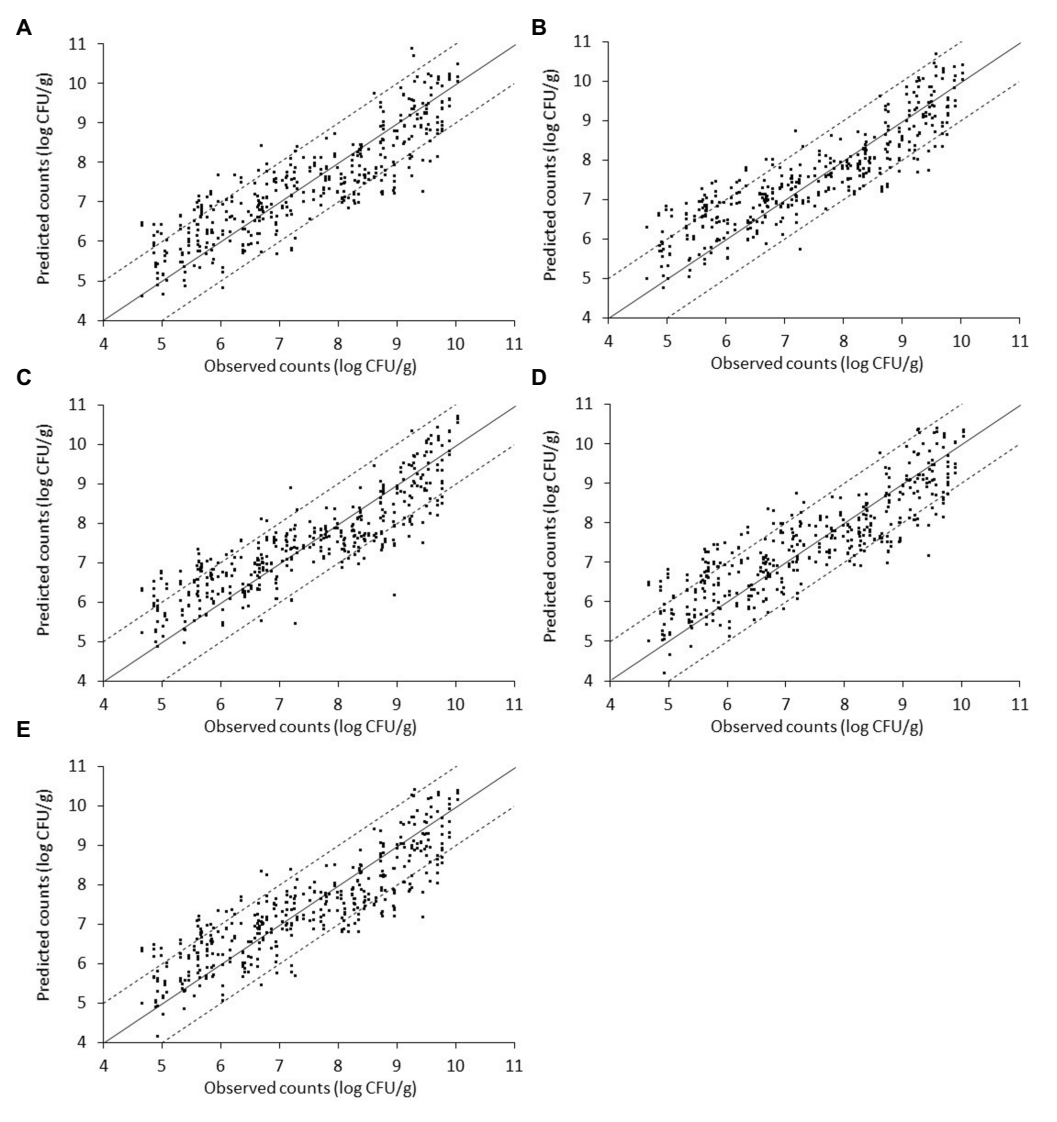
Scatterplot of the microbial populations measured *via* microbiological analysis and estimated by the support vector machine regression model (external validation) based on Fourier-transform infrared spectra from non-inoculated liver. **(A)** TVC **(B)** *Pseudomonas* spp. **(C)** *Brochothrix thermosphacta* **(D)** lactic acid bacteria, and **(E)** *Enterobacteriaceae* (solid line: the ideal y = x line; dashed lines: ±1 log unit area). Data points from 50 iterations are presented.

**Figure 8:**
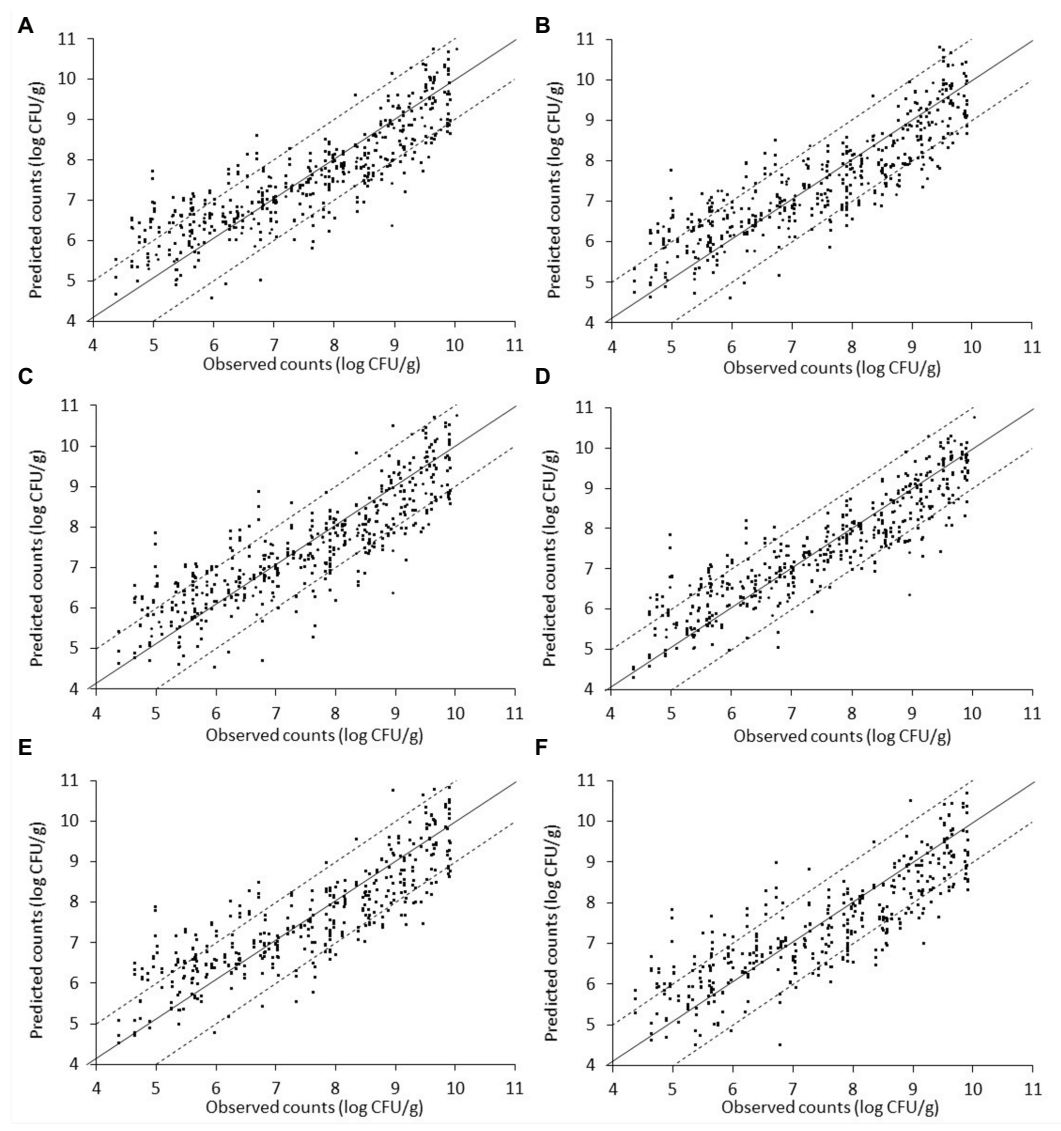
Scatterplot of the microbial populations measured *via* microbiological analysis and estimated by the support vector machine regression model (external validation) based on Fourier-transform infrared spectra from liver inoculated with *Salmonella*. **(A)** TVC **(B)** *Pseudomonas* spp. **(C)** *Brochothrix thermosphacta* **(D)** lactic acid bacteria **(E)** *Enterobacteriaceae*, and **(F)** *Salmonella* (solid line: the ideal y = x line; dashed lines: ±1log unit area). Data points from 50 iterations are presented.

**Figure 9:**
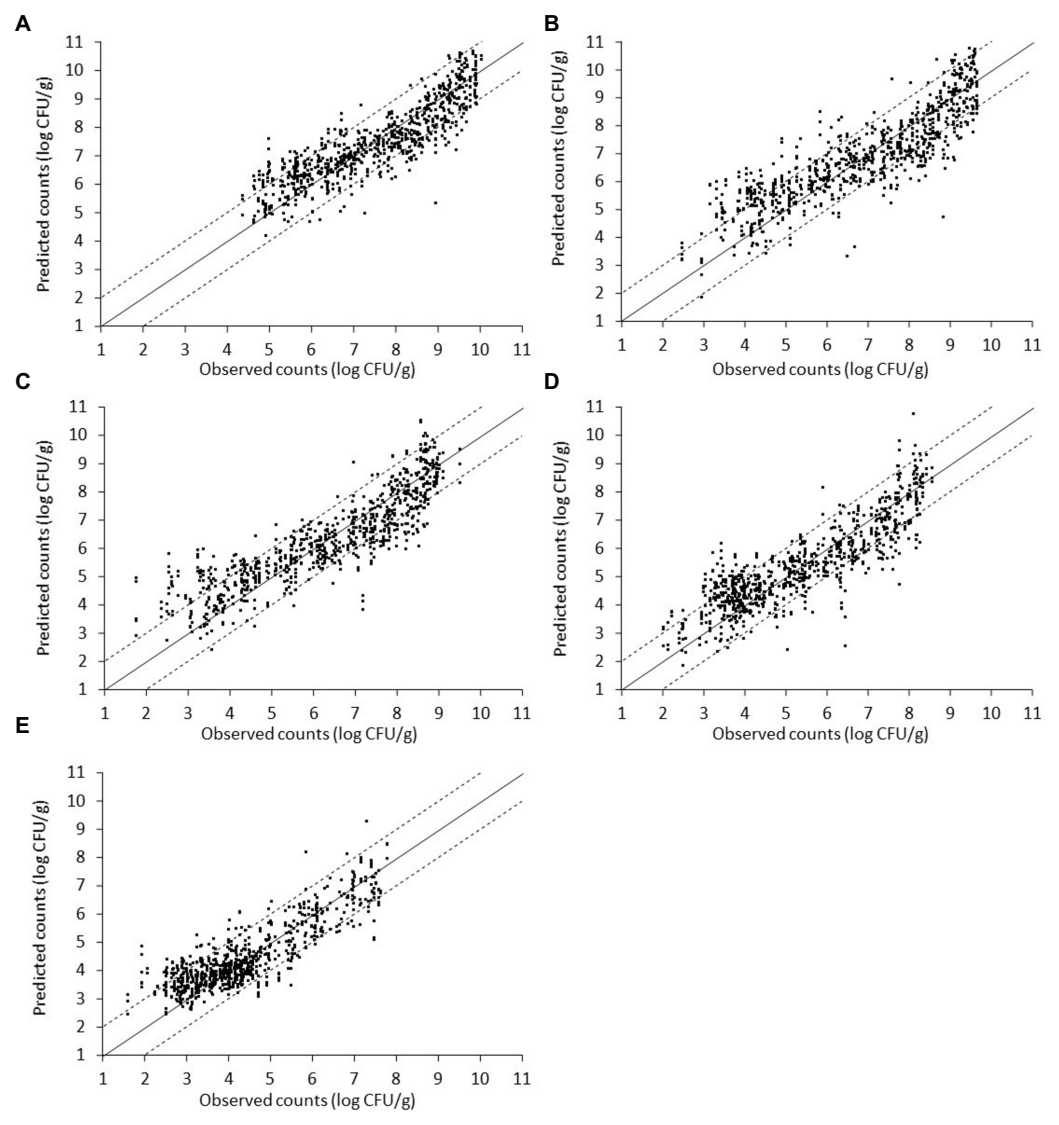
Scatterplot of the microbial populations measured *via* microbiological analysis and estimated by the support vector machine regression model (external validation) based on combined Fourier-transform infrared spectra (i.e., from non-inoculated and inoculated with *Salmonella* liver). **(A)** TVC, **(B)** *Pseudomonas* spp., **(C)** *Brochothrix thermosphacta*, **(D)** lactic acid bacteria, and **(E)** *Enterobacteriaceae* (solid line: the ideal y = x line; dashed lines: ±1 log unit area). Data points from 50 iterations are presented.

The relation between the measured (*via* microbiological analysis) and the estimated (by the model) microbial populations on chicken liver is illustrated in Figures 7–9. The predictive reliability and accuracy of the developed models for the different bacterial groups in the three datasets, i.e., from non-inoculated, inoculated with *Salmonella*, and their combination, were evaluated by calculating different statistical metrics (Tables 1–3). In the case of non-inoculated liver samples, the plot of the observed and predicted counts presented positive association and good distribution around the line of equity without any particular trend, with more than 80% of predictions (ranging from 80.49 to 82.52%, depending on the estimated microbial group) within the ±1 log unit area of the actually observed ones (Figure 7, Table 1). The bias factor B*_f_* was generally very close to unity (ranging from 0.995 to 1.022, depending on the microbial group), indicating no structural deviation of the models, i.e., systematic overprediction (B*_f_* > 1) or underprediction (B*_f_* < 1; Table 1; Koutsoumanis et al., 2006; Oscar, 2009). Actually, B*_f_* values within the range of 0.9–1.05 are considered as adequate in model development (Ross, 1999; Ross et al., 2000). In addition, the value of accuracy factor A*_f_* indicated that the average deviation between predictions and observations was 8.4% (either below or above the line of equity) for *Pseudomonas* spp. and *Enterobacteriaceae*, 8.5% for *B. thermosphacta*, 8.6% for LAB, and 8.7% for TVC (Table 1). It has been suggested that A*_f_* values with an increase 0.15 (15%) are considered as satisfactory in models employing only one variable, which is the case of this study (Ross et al., 2000). The calculated *R*^2^ and RMSE metrics were found to be similar for all the microbial groups predicted, ranging from 0.768 to 0.791 and 0.698 to 0.733 log CFU/g, respectively, indicating a good model performance.

**Table 1:** Performance metrics of the support vector machine models for the prediction of the different microbial populations of chicken liver (non-inoculated) stored under aerobic conditions at isothermal and dynamically changing conditions.

Metrics	Total viable counts	*Pseudomonas* spp.	*Brochothrix thermosphacta*	Lactic acid bacteria	*Enterobacteriaceae*
	Mean ± SD^a^	Mean ± SD	Mean ± SD	Mean ± SD	Mean ± SD
	(upper, lower CI)	(upper, lower CI)	(upper, lower CI)	(upper, lower CI)	(upper, lower CI)
*R*^2^	0.769 ± 0.022	0.791 ± 0.017	0.776 ± 0.024	0.768 ± 0.027	0.778 ± 0.020
	(0.763, 0.775)	(0.786, 0.795)	(0.769, 0.783)	(0.760, 0.775)	(0.772, 0.783)
RMSE	0.729 ± 0.035	0.698 ± 0.022	0.721 ± 0.035	0.733 ± 0.035	0.716 ± 0.030
	(0.719, 0.738)	(0.692, 0.704)	(0.711, 0.730)	(0.723, 0.743)	(0.708, 0.724)
A*_f_*	1.087 ± 0.005	1.084 ± 0.004	1.085 ± 0.004	1.086 ± 0.005	1.084 ± 0.004
	(1.085, 1.088)	(1.083, 1.086)	(1.084, 1.086)	(1.085, 1.087)	(1.083, 1.085)
B*_f_*	1.003 ± 0.006	1.022 ± 0.008	1.011 ± 0.006	1.011 ± 0.008	0.995 ± 0.006
	(1.001, 1.004)	(1.020, 1.024)	(1.009, 1.013)	(1.009, 1.014)	(0.994, 0.997)
Accuracy (%)	80.653 ± 3.043	82.517 ± 1.950	80.490 ± 2.747	80.571 ± 2.804	81.088 ± 2.847
	(79.810, 81.496)	(81.977, 83.057)	(79.728, 81.251)	(79.794, 8 1.349)	(80.299, 81.877)

a Total of 50 iterations.

CI, 95% confidence interval; *R*^2^, coefficient of determination; RMSE, root mean square error (log CFU/g); A*_f_*, accuracy factor; B*_f_*, bias factor.

**Table 2:** Performance metrics of the support vector machine models for the prediction of the different microbial populations of chicken liver inoculated with *Salmonella* stored under aerobic conditions at isothermal and dynamically changing conditions.

Metrics	Total viable counts	*Pseudomonas* spp.	*Brochothrix thermosphacta*	Lactic acid bacteria	*Enterobacteriaceae*	*Salmonella*
	Mean ± SD^a^	Mean ± SD	Mean ± SD	Mean ± SD	Mean ± SD	Mean ± SD
	(upper, lower CI)	(upper, lower CI)	(upper, lower CI)	(upper, lower CI)	(upper, lower CI)	(upper, lower CI)
*R*^2^	0.728 ± 0.027	0.730 ± 0.031	0.725 ± 0.028	0.828 ± 0.020	0.719 ± 0.036	0.708 ± 0.027
	(0.721, 0.735)	(0.722, 0.739)	(0.717, 0.733)	(0.822, 0.833)	(0.709, 0.729)	(0.701, 0.716)
RMSE	0.837 ± 0.040	0.827 ± 0.051	0.841 ± 0.049	0.664 ± 0.039	0.847 ± 0.053	0.863 ± 0.044
	(0.826, 0.847)	(0.813, 0.841)	(0.828, 0.855)	(0.653, 0.675)	(0.832, 0.861)	(0.851, 0.875)
A*_f_*	1.098 ± 0.005	1.095 ± 0.007	1.096 ± 0.006	1.071 ± 0.005	1.095 ± 0.007	1.102 ± 0.005
	(1.097, 1.100)	(1.093, 1.097)	(1.095, 1.098)	(1.070, 1.073)	(1.093, 1.097)	(1.100, 1.103)
B*_f_*	1.017 ± 0.009	1.012 ± 0.008	1.008 ± 0.009	1.005 ± 0.007	1.016 ± 0.009	0.995 ± 0.007
	(1.014, 1.019)	(1.009, 1.014)	(1.005, 1.010)	(1.003, 1.007)	(1.014, 1.019)	(0.993, 0.997)
Accuracy (%)	74.234 ± 2.733	73.572 ± 3.239	75.338 ± 2.977	85.669 ± 2.667	75.090 ± 3.086	73.076 ± 2.603
	(73.477, 74.992)	(72.675, 74.470)	(74.513, 76.163)	(84.930, 86.408)	(74.234, 75.945)	(72.354, 73.797)

a Total of 50 iterations.

CI, 95% confidence interval; *R*^2^, coefficient of determination; RMSE, root mean square error (log CFU/g); A*_f_*, accuracy factor; B*_f_*, bias factor.

**Table 3:** Performance metrics of the support vector machine models for the prediction of the different microbial populations of chicken liver based on a combination of spectral data from non-inoculated and inoculated-with-*Salmonella* chicken liver samples.

Metrics	Total viable counts	*Pseudomonas* spp.	*Brochothrix thermosphacta*	Lactic acid bacteria	*Enterobacteriaceae*
	Mean ± SD^a^	Mean ± SD	Mean ± SD	Mean ± SD	Mean ± SD
	(upper, lower CI)	(upper, lower CI)	(upper, lower CI)	(upper, lower CI)	(upper, lower CI)
*R*^2^	0.775 ± 0.022	0.766 ± 0.020	0.797 ± 0.017	0.741 ± 0.024	0.737 ± 0.023
	(0.769, 0.781)	(0.760, 0.772)	(0.792, 0.802)	(0.734, 0.747)	(0.730, 0.743)
RMSE	0.743 ± 0.032	0.949 ± 0.040	0.888 ± 0.042	0.849 ± 0.036	0.696 ± 0.025
	(0.735, 0.752)	(0.938, 0.960)	(0.877, 0.900)	(0.839, 0.859)	(0.689, 0.703)
A*_f_*	1.083 ± 0.004	1.126 ± 0.007	1.132 ± 0.007	1.145 ± 0.007	1.141 ± 0.007
	(1.082, 1.084)	(1.125, 1.128)	(1.130, 1.134)	(1.143, 1.147)	(1.140, 1.143)
B*_f_*	0.999 ± 0.006	1.020 ± 0.008	1.020 ± 0.008	1.009 ± 0.007	1.029 ± 0.009
	(0.997, 1.001)	(1.018, 1.022)	(1.018, 1.023)	(1.007, 1.011)	(1.026, 1.031)
Accuracy (%)	80.840 ± 1.735	71.761 ± 2.117	76.546 ± 2.043	77.884 ± 1.898	84.334 ± 1.681
	(80.359, 81.320)	(71.174, 72.348)	(75.980, 77.112)	(77.358, 78.410)	(83.869, 84.800)

a Total of 50 iterations.

CI, 95% confidence interval; *R*^2^, coefficient of determination; RMSE, root mean square error (log CFU/g); A*_f_*, accuracy factor; B*_f_*, bias factor.

The relation between observed and predicted values appeared slightly inferior in the case of inoculated-with-*Salmonella* samples compared to the non-inoculated samples (Figure 8). However, based on related plots in Figure 8, predictions vs. observations again presented a relatively good distribution around the line of equity, with more than 73% of the data included within the ±1 log unit area. Briefly, accuracy (%) varied from 73.08 to 85.67% in descending order for microbial populations of LAB, *B. thermosphacta*, *Enterobacteriaceae*, TVC, *Pseudomonas* spp., and *Salmonella* (Figure 8, Table 2). With regards to B*_f_*, analogous results were obtained, with values ranging from 0.995 to 1.017 for the different bacterial groups, signifying an optimum correlation between observed and predicted values (Ross et al., 2000; Argyri et al., 2010). On the other hand, A*_f_* was close to 1, indicating that predictions were close to observations, and ranged from 7.1% for LAB to 9.5% for *Pseudomonas* spp. and *Enterobacteriaceae*, 9.6% for *B. thermosphacta*, and 10.2% for *Salmonella*. The calculated *R*^2^ and RMSE values for the different microbial groups predicted ranged from 0.708 to 0.828 and 0.664 to 0.863 log CFU/g, respectively, with a slightly better performance observed in the case of LAB. Models derived from inoculated samples had a comparable performance with models derived from non-inoculated ones.

Finally, spectral and microbiological data from the combination of non-inoculated and inoculated-with-*Salmonella* samples were employed into the analysis. In this case, the input dataset consisted of a random mixture of non-inoculated and inoculated-with-*Salmonella* samples in order to evaluate the potential of the developed pipeline in achieving a good prediction accuracy regardless of the presence or absence of the pathogen. Plots of observed vs. predicted microbial counts presented a reasonably good distribution around the equity line, close to that observed for non-inoculated data (Figure 9). As can be visualized from Figure 9 and Table 3, approximately 84.33, 80.84, 77.88, 76.55, and 71.76% of the predicted microbial counts for *Enterobacteriaceae*, TVC, LAB, *B. thermosphacta*, and *Pseudomonas* spp., respectively, were within the ±1 log unit area. The B*_f_* was close to 1 (ranging from 0.999 to 1.029) for all bacterial groups, while A*_f_* was 1.083 for TVC, 1.126 for *Pseudomonas* spp., 1.132 for *B. thermosphacta*, 1.145 for *Enterobacteriaceae*, and 1.141 for LAB. The calculated *R*^2^ and RMSE values ranged from 0.737 to 0.797 and 0.696 to 0.949 log CFU/g, respectively, suggesting a satisfactory relationship between spectra and the specific spoilage organisms studied.

Available research data on the potential of FTIR spectroscopy to determine the microbiological spoilage on the surface of chicken products are relatively limited. Ellis et al. (2002) employed a partial least squares (PLS) regression model to accurately estimate (RMSE of 0.27 log CFU/g) TVC populations on chicken breasts during storage at room temperature. PLS regression was also carried out by Vasconcelos et al. (2014) to determine specific microorganisms on chicken breast such as *Pseudomonas* spp., LAB, *Enterobacteriaceae*, and *B. thermosphacta* from related FTIR spectra, obtaining *R*^2^ of 0.789, 0.832, 0.857, and 0.810, respectively. Slightly less accurate results were observed by Rahman et al. (2018), who developed a PLS model to predict total plate count (TPC) and *Enterobacteriaceae* on chicken breast surfaces during aerobic refrigerated storage, generating a good performance with *R*^2^ being 0.66 for TPC and 0.52 for *Enterobacteriaceae*, respectively. However, to our knowledge, this is the first study reporting the considerable potential of FTIR spectroscopy in rapid and non-destructive quantitative assessment of microbiological spoilage on chicken liver.

## Conclusion

Chicken liver constitutes a highly perishable food commodity due to the relatively high contents of readily available nutrients and water activity that support microbial growth. In this context, the growth of indigenous spoilage microbiota and the behavior of inoculated *Salmonella* on chicken liver stored aerobically under refrigeration (isothermal and dynamically changing temperatures) were initially investigated. Spoilage was mainly attributed to the presence of *Pseudomonas* spp. as well as of *B. thermosphacta*, followed by LAB and *Enterobacteriaceae*, while the contribution of yeasts/molds was limited. Microbiological spoilage was affected by the storage temperature as well as the inherent and microbiological variability observed on chicken liver (fresh and/or spoiled). Chicken liver supported the survival of inoculated *Salmonella* at 0°C and, most importantly, its growth at 4 and 8°C, indicating the need for the application of sanitation and safe food handling procedures.

Furthermore, the ability of FTIR spectroscopy to estimate the populations of TVC, *Pseudomonas* spp., *B. thermosphacta*, *Enterobacteriaceae*, LAB, and *Salmonella* on chicken liver was explored. The proposed pipeline incorporated the inherent (batch) variability of chicken liver samples, the variability of storage temperature (isothermal and dynamically changing temperatures), and the biochemical fingerprint of *Salmonella* in the event of cross-contamination. The results of the current study outline the efficiency of FTIR spectroscopy in tandem with the described data analysis and model building workflow to satisfactorily describe spoilage on chicken liver.

## Data Availability Statement

The raw data supporting the conclusions of this article will be made available by the authors, without undue reservation.

## Author Contributions

AA and CT contributed to the conceptualization of this study. The methodology was an equal contribution of DD and AG. DD, AG, and GF conducted the validation. DD, AG, AA, AD, and GF performed the formal analysis. CT and G-JN took charge of the resources. DD, AA, and PT were in charge of data curation. DD and AG were responsible for the original draft preparation. DD, AA, AD, NC, and CT reviewed and edited the manuscript. AA, G-JN, and CT supervised the study. CT was in charge of project administration. All authors contributed to the article and approved the submitted version.

### Conflict of Interest

The authors declare that the research was conducted in the absence of any commercial or financial relationships that could be construed as a potential conflict of interest.
